# Affibody-Based PET Imaging to Guide EGFR-Targeted Cancer Therapy in Head and Neck Squamous Cell Cancer Models

**DOI:** 10.2967/jnumed.118.216069

**Published:** 2019-03

**Authors:** Thomas A. Burley, Chiara Da Pieve, Carlos D. Martins, Daniela M. Ciobota, Louis Allott, Wim J.G Oyen, Kevin J. Harrington, Graham Smith, Gabriela Kramer-Marek

**Affiliations:** 1 Division of Radiotherapy and Imaging, Institute of Cancer Research, London, United Kingdom; and; 2 Department of Nuclear Medicine, Royal Marsden NHS Foundation Trust, London, United Kingdom

**Keywords:** Affibody molecules, EGFR, ^89^Zr, ^18^F, cancer imaging

## Abstract

In head and neck squamous cell cancer, the human epidermal growth factor receptor 1 (EGFR) is the dominant signaling molecule among all members of the family. So far, cetuximab is the only approved anti-EGFR monoclonal antibody used for the treatment of head and neck squamous cell cancer, but despite the benefits of adding it to standard treatment regimens, attempts to define a predictive biomarker to stratify patients for cetuximab treatment have been unsuccessful. We hypothesized that imaging with EGFR-specific radioligands may facilitate noninvasive measurement of EGFR expression across the entire tumor burden and allow for dynamic monitoring of cetuximab-mediated changes in receptor expression. **Methods:** EGFR-specific Affibody molecule (Z_EGFR:03115_) was radiolabeled with ^89^Zr and ^18^F. The radioligands were characterized in vitro and in mice bearing subcutaneous tumors with varying levels of EGFR expression. The protein dose for imaging studies was assessed by injecting ^89^Zr-deferoxamine-Z_EGFR:03115_ (2.4–3.6 MBq, 2 μg) either together with or 30 min after increasing amounts of unlabeled Z_EGFR:03115_ (1, 5, 10, 15, and 20 μg). PET images were acquired at 3, 24, and 48 h after injection, and the image quantification data were correlated with the biodistribution results. The EGFR expression and biodistribution of the tracer were assessed ex vivo by immunohistochemistry, Western blot, and autoradiography. To downregulate the EGFR level, treatment with cetuximab was performed, and ^18^F-aluminium fluoride-NOTA-Z_EGFR:03115_ (12 μg, 1.5–2 MBq/mouse) was used to monitor receptor changes. **Results:** In vivo studies demonstrated that coinjecting 10 μg of nonlabeled molecules with ^89^Zr-deferoxamine-Z_EGFR:03115_ allows for clear tumor visualization 3 h after injection. The radioconjugate tumor accumulation was EGFR-specific, and PET imaging data showed a clear differentiation between xenografts with varying EGFR expression levels. A strong correlation was observed between PET analysis, ex vivo estimates of tracer concentration, and receptor expression in tumor tissues. Additionally, ^18^F-aluminium fluoride-NOTA-Z_EGFR:03115_ could measure receptor downregulation in response to EGFR inhibition. **Conclusion:** Z_EGFR:03115_-based radioconjugates can assess different levels of EGFR level in vivo and measure receptor expression changes in response to cetuximab, indicating a potential for assessment of adequate treatment dosing with anti-EGFR antibodies.

Globally, head and neck squamous cell cancer (HNSCC) is the sixth most common cancer; its treatment consists of surgery or radiotherapy, with or without concurrent chemotherapy or targeted therapy (1). Disappointingly, patient survival has not markedly improved in recent decades, and approximately 50% of patients with locally advanced disease will develop recurrence or metastases within 2 y. Such differences in outcome are largely driven by inter- and intrapatient heterogeneity in disease biology (2).

Of note, in HNSCC the human epidermal growth factor receptor 1 (EGFR) is the dominant signaling molecule among all members of the family (3). EGFR messenger RNA and receptor expression were found to be elevated in 92% and 38%–47% of cases, respectively (3,4). Furthermore, this aberrant receptor expression and activity were positively correlated with poor patient prognosis and resistance to radiation therapy (5). These findings led to the development and widespread implementation of specific anti-EGFR inhibitors, including monoclonal antibodies (mAbs) targeting the extracellular domain of EGFR (e.g., cetuximab, panitumumab, and zalutumumab) and small-molecule tyrosine kinase inhibitors targeting the intracellular domain (e.g., gefitinib and erlotinib) (6). So far, cetuximab is the only anti-EGFR therapy agent approved for the treatment of HNSCC. However, despite the clearly documented clinical benefits of cetuximab, attempts to define a predictive biomarker to stratify patients for treatment with the antibody have been unsuccessful. Remarkably, measurement of levels of EGFR protein expression or receptor activation (e.g., the presence of activating gene mutations) has not demonstrated any predictive value benefit to cetuximab treatment in HNSCC. In the context of radical, curative radiotherapy or chemoradiotherapy, dichotomizing EGFR expression into low versus high levels (defined as <50% or >50% positive cells in a clinical trial with radiotherapy and <80% or >80% positive cells in the RTOG0522 study with chemoradiotherapy) did not predict benefit from the addition of cetuximab (2,7,8). Similarly, in the EXTREME trial of first-line use of platin/5-fluorouracil/cetuximab in relapsed or metastatic disease, outcomes were essentially identical for patients with less than or more than 40% EGFR-positive tumor cells (9). As a consequence, at present the clinical use of cetuximab is not based on the measurement of intratumoral EGFR expression or, indeed, of any other biomarker. Against this background, it is important to consider the context in which EGFR has been assessed in previous clinical studies, in order to understand the limitations of the methodologies used. At this moment, no serum biomarker has been identified that can consistently classify an EGFR-positive subgroup of patients for targeted therapies; therefore, in most clinical trials, EGFR expression has been measured via immunohistochemical staining before the initiation of therapy at a single, static time point, and on a relatively small tissue sample (10). For patients with relapsed or metastatic disease, the assessment may have been performed on archival specimens and, furthermore, the reported immunohistochemistry data are subject to variability in the sensitivity and specificity of reagents, the use of different staining protocols, and concerns relating to the tissue sample that could have provided an unrepresentative view of heterogeneous receptor expression. Therefore, we hypothesized that incorporating imaging with EGFR-specific radioligands into routine clinical practice may not only facilitate a noninvasive, real-time measurement of EGFR expression across the patient’s entire tumor burden but also allow for dynamic monitoring of cetuximab-mediated receptor downregulation, providing a marker for adequate treatment dosing.

To date, the use of EGFR-specific molecular imaging for HNSCC patients has been limited to radiolabeled mAbs. Even though they have demonstrated high uptake in EGFR-positive tumors, their large molecular size (∼150 kDa) and consequential slow hepatobiliary clearance (e.g., cetuximab half-life in humans is about 95 h) resulted in poor contrast between tumor and normal tissues on images acquired at earlier time points (11,12). Accordingly, using smaller molecules, such as mAb fragments (e.g., F(ab′)_2_, Fab′, single-domain antibodies [∼15 kDa], or Affibody [Affibody AB] scaffolds [∼7 kDa]) that are characterized by rapid tumor penetration and fast blood clearance could facilitate high-contrast imaging as early as a few hours after injection (13). Indeed, these favorable pharmacokinetics have been demonstrated by PET imaging of EGFR-expressing xenografts using EGFR-specific Affibody molecules conjugated to several radioisotopes, such as ^18^F (14,15), ^89^Zr (16,17), and ^11^C (18). Of note, a study by Garousi et al. recently showed greater tumor-to-blood ratios 3 h after administration of ^89^Zr-deferoxamine (DFO)-Z_EGFR:2377_ than 48 h after injection of ^89^Zr-DFO-cetuximab, highlighting the advantage of imaging with smaller molecules (19).

Herein, we report the use of a radiolabeled Affibody molecule (Z_EGFR:03115_) to noninvasively measure differences in EGFR expression, both in vitro and in subcutaneous HNSCC xenograft models. An ^89^Zr-labeled conjugate was used to assess tumor-to-organ ratios at different time points and a ^18^F-labeled analog to measure the response to cetuximab treatment in vivo. These data support the hypothesis that a targeted PET agent can quantify EGFR expression level and may represent a powerful missing tool that will facilitate informed image-guided anti-EGFR therapeutic strategies in the clinic.

## MATERIALS AND METHODS

### Cell Lines

Human head and neck cancer cell line HN5 (EGFR++++) was provided by Prof. Kevin Harrington (Institute of Cancer Research); CAL27 (EGFR+++), Detroit562 (EGFR++), and human breast adenocarcinoma cell line MCF7 (EGFR+) were purchased from the American Type Culture Collection. Cells were cultured in Dulbecco modified Eagle medium (Gibco, Life Technologies) supplemented with 10% heat-inactivated fetal bovine serum (Gibco, Life Technologies) and grown as monolayers at 37°C in a humidified atmosphere containing 5% CO_2_.

### Preparation of Z_EGFR:03115_-DyLight633, ^89^Zr-DFO- and ^18^F-Aluminium Fluoride (AlF)-NOTA Affibody Conjugates

The conjugation of DyLight633, DFO-maleimide, and maleimidoethylmonoamide NOTA to the Affibody molecules and the consequent ^89^Zr and ^18^F-AlF radiolabeling procedures are described in the Supplemental Data (supplemental materials are available at http://jnm.snmjournals.org).

### Flow Cytometry Analysis of EGFR Expression

To assess the EGFR expression in the HN5, CAL27, Detroit562, and MCF7 cell lines, samples were incubated for 1 h at 4°C with a fluorescein isothiocyanate–conjugated EGFR-specific antibody (20 nM) (Santa Cruz Biotechnology). Afterward, the cells were washed in phosphate-buffered saline and analyzed on the BD LSRII flow cytometer (Becton Dickinson). The results were analyzed using FlowJo, version 10 (FlowJo, LLC).

### Confocal Microscopy

The binding specificity of the Affibody molecule to EGFR was assessed by incubating HN5 cells with Z_EGFR:03115_-Dylight633 (1 μM) for 1 h at 37°C with and without preincubation with a 100-fold excess of the Z_EGFR:03115_. To study internalization of the conjugate, HN5 cells were incubated with Z_EGFR:03115_-Dylight633 (1 μM) for 1 h at 37°C and washed with phosphate-buffered saline, and confocal images were acquired at 3, 8, and 24 h after incubation. All cells were counterstained with Hoechst 33342 (nuclear stain; Thermo Fisher Scientific) and Lysotracker Green DND-26 (lysosome stain; Thermo Fisher Scientific) 1 h before the images were captured using the LSM700 confocal microscope (Carl Zeiss Inc.). Images (8 bit, 1,024 × 1,024) were analyzed using Zen software, version 2009 (Zeiss).

### Immunoblotting

Western blotting was performed as previously described in the literature (20). Briefly, the proteins were immunoblotted with primary antibodies against EGFR, p-EGFR (Tyr1068), AKT (protein kinase B), p-AKT (S473), β-actin, or GAPDH (all from Cell Signaling Technology) overnight at 4°C. On the following day, membranes were rinsed and incubated with horseradish peroxidase–conjugated secondary antibodies (Cell Signaling Technology) for 1 h at room temperature. The immunoblots were visualized after the addition of SuperSignal West Pico Plus Chemiluminescent Substrate (Thermo Fisher Scientific) and imaged with a ChemiDoc XRS+ System (Bio-Rad). The densitometry was performed using ImageJ (NIH).

### In Vitro Binding Affinity and Specificity

The disassociation constants (K_d_) of ^89^Zr- and ^18^F-AlF–based Z_EGFR:03115_ conjugates were assessed by a saturation binding assay. The CAL27 cells were incubated with increasing concentrations of radiolabeled Z_EGFR:03115_ (0.1–50 nM) for 2 h at 4°C. Nonspecific binding was determined by adding a 100-fold excess of unlabeled Z_EGFR:03115_. Cell-bound radioactivity was determined using the 2480WIZARD^2^ automatic γ-counter (PerkinElmer). To estimate the K_d_, the data were plotted as the concentration of bound labeled Affibody (nM) versus concentration of the labeled Affibody added (nM). The specific binding was measured by subtracting the fraction of the nonspecific from the total binding and fitted using a nonlinear regression curve and a 1-site specific receptor-binding model using GraphPad Prism software, version 7.0.

To evaluate the binding specificity of Z_EGFR:03115_, we incubated HN5, CAL27, Detroit562, and MCF7 cells with either ^89^Zr-DFO-Z_EGFR:03115_ or ^18^F-AlF-NOTA-Z_EGFR:03115_ (20 nM) for 1 h at 4°C. To some of the cells, a 100-fold excess of either cetuximab (Merck), natural EGF ligand (Thermo Fisher Scientific), or unlabeled Z_EGFR:03115_ was added. Afterward, the cells were rinsed and trypsinized, and the radioactivity was measured using a γ-counter. Each experiment was normalized to the maximum cell-associated radioactivity and presented as the mean of 3 independent experiments (performed in triplicate) ± SEM.

### Biodistribution

All experiments were performed in compliance with licenses issued under the U.K. Animals (Scientific Procedures) Act of 1986. Studies were compliant with the U.K. National Cancer Research Institute Guidelines for Animal Welfare in Cancer Research (21). Female NU(NCr)-Foxn1^nu^ mice were purchased from Charles River Laboratories. To generate tumor xenografts, CAL27 (4 × 10^6^), Detroit562 (6 × 10^6^), or MCF7 (6.5 × 10^6^) cells were inoculated in BD Matrigel matrix subcutaneously into the shoulder of the mouse. For the MCF7 xenograft model, a 17β-estradiol pellet (0.72 mg, 90-d release) was implanted in each mouse 48 h before cell inoculation. The tumors were allowed to grow until reaching an approximate volume of 100 mm^3^.

Dose-dependent ^89^Zr-DFO-Z_EGFR:03115_ uptake was assessed by injecting the ^89^Zr-DFO- Z_EGFR:03115_ into the tail vein (2 μg) either together with or 30 min after increasing amounts of unlabeled Affibody molecule (1, 5, 10, 15, and 20 μg). ^89^Zr-Z_Taq_ (Taq DNA polymerase-specific Affibody molecule) (3–3.1 MBq, 2 μg) was used as a negative control. Animals were killed at 3, 24, and 48 h after injection. Blood was collected, the major organs were excised and weighed, and their associated radioactivity was measured using a γ-counter. Unless otherwise stated, the results for each tissue sample were calculated as percentage injected dose per gram of tissue (%ID/g) (*n* ≥ 3 mice ± SD).

### Autoradiography

Dissected tumors were embedded in an optimal-cutting-temperature compound (Tissue-Tek; Sakura) and immediately snap-frozen on dry ice. The specimens were sectioned to a thickness of 6 μm using a cryomicrotome (Thermo Fisher Scientific) and mounted on slides. The slides were exposed to a photostimulable phosphor plate for 24 h and read using a Typhoon7000 phosphor imager (GE Healthcare Life Sciences). The recorded images were analyzed using ImageQuant TL, version 8.1 (GE Healthcare Life Sciences).

### Immunohistochemistry

The tumors were fixed in formalin and were paraffin-embedded to be sectioned and further processed (Breast Cancer Now Histopathology Core Facility). Multiple sections were taken at regular intervals across each tumor, stained with hematoxylin and eosin (Leica) and an anti-EGFR mAb (Dako pharmDx) according to the manufacturer’s protocol, and imaged with the NanoZoomer-XR (Hamamatsu Photonics).

### PET Imaging

The mice were anesthetized using isoflurane (1.5%–2% v/v in O_2_) before imaging. Using an Albira PET/SPECT/CT preclinical imaging system (Bruker), whole-body 15-min (^89^Zr) or 10-min (^18^F) static images were acquired with an energy window of 358–664 keV, followed by CT acquisition. PET data were reconstructed using a maximum-likelihood expectation-maximization algorithm (12 iterations), and scatter and attenuation corrections were applied using their respective CT scans. High-resolution CT scans were performed with the x-ray tube set up at a voltage of 45 kV, a current of 400 μA, 250 projections (1 s per projection), and a voxel size of 0.5 × 0.5 × 0.5 mm. The CT images were reconstructed using a filtered-backprojection algorithm. The PMOD software package (PMOD Technologies Ltd.) was used to analyze the images. The tumor volume was selected by first drawing volumes of interest around the tumor and selecting a 50% maximum pixel isocontour. The mean counts were extracted and converted into %ID/g using a calibration factor (MBq/g/counts) calculated by scanning a source (^89^Zr or ^18^F) of known activity and volume.

### Cetuximab Treatment

Cetuximab treatment studies were conducted using HN5 xenografts, as this model is particularly sensitive to cetuximab treatment (22). Mice were randomized into either control (*n* = 6) or treatment (*n* = 7) groups and treated with a vehicle or cetuximab, respectively. The control mice were injected with ^18^F-AlF-NOTA-Z_EGFR:03115_ (12 μg, 1.5–2 MBq/mouse), imaged 1 h after conjugate administration, and killed for biodistribution study. Mice from the treatment group underwent cetuximab treatment (a 600-μg bolus injection every 3 d for a total of 4 doses delivered via intraperitoneal injection). Allowing 13 d for posttreatment clearance of the mAb, subsequent PET images were acquired with ^18^F-AlF-NOTA-Z_EGFR:03115_ (12 μg, 1.5–2 MBq/mouse). In addition, to demonstrate a lack of competition between the Affibody and the cetuximab remaining in the blood circulation, an extra group of mice (*n* = 3) was coinjected with cetuximab (3.75 μg, 26 pmol)—an amount that was estimated to still be present in the blood 13 d after completion of treatment, considering a mAb biologic half-life of 40 h. Densitometric analysis of the tumor tissue lysates was performed using ImageJ (NIH). Data are presented as mean ± SEM (blots, *n* = 2).

### Statistical Analysis

Statistical analyses were performed Prism software.

Significance was determined using the unpaired 2-tailed Student *t* test with Welch correction. To determine statistical significance between uptake in the different xenograft models, a 2-way ANOVA with Tukey correction was used. Correlation analysis was performed using Spearman rank correlation, with linear regression and 95% confidence intervals. Statistically significant differences between groups were assumed if the *P* value was 0.05 or less. No data were excluded from the analysis.

## RESULTS

### Z_EGFR:03115_ Targeting Properties

**FIGURE 1.**
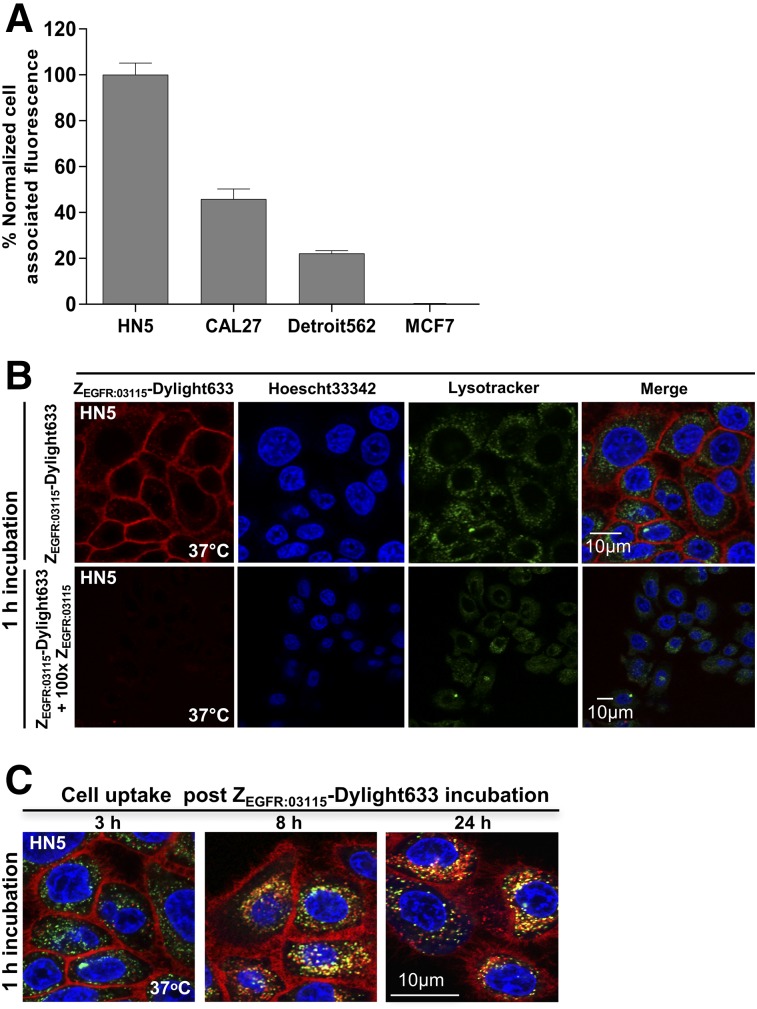
(A) EGFR expression as determined by flow cytometry in selected cancer cell lines. (B) In vitro binding specificity of Z_EGFR:03115_-Dylight633 in HN5 cells as shown by confocal microscopy. (C) Internalization studies of Z_EGFR:03115_-Dylight633 3, 8, and 24 h after 1 h of incubation in HN5 cells.

Initially, EGFR expression was determined in the selected cancer cell lines by Western blotting and flow cytometry. The Western blotting results revealed varying levels of EGFR expression, from high, HN5 (EGFR++++); to medium, CAL27 (EGFR+++); to low, Detroit562 (EGFR++); to negligible, MCF7 (EGFR+) (Supplemental Fig. 1), as was concordant with the flow cytometry data using Z_EGFR:03115_-Dylight633 (Fig. 1A).

To demonstrate that Z_EGFR:03115_ specifically targets EGFR, HN5 cells were incubated with Z_EGFR:03115_-Dylight633 and the cell-associated fluorescence was visualized by confocal microscopy. After a 1-h incubation at 37°C, an intense fluorescent signal was found on the cell membrane (Fig. 1B). Images acquired 3, 8, and 24 h later showed an intracellular accumulation of the conjugate, although most still remained bound to the membrane, even 24 h after incubation (Fig. 1C). Additionally, incubating the cells with a 100-fold excess of unlabeled Z_EGFR:03115_ markedly decreased the fluorescent signal (Fig. 1B, bottom), demonstrating that binding is EGFR-specific and receptor-mediated.

### In Vitro Binding of ^89^Zr-DFO-Z_EGFR:03115_

**FIGURE 2.**
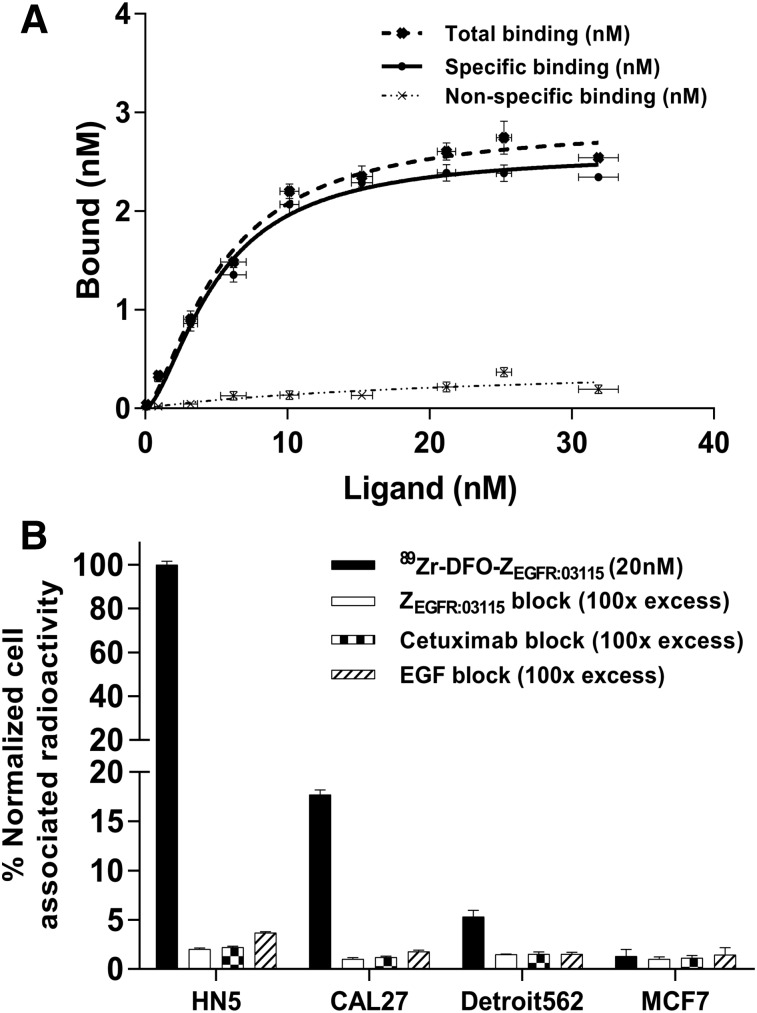
(A) Saturation curve obtained for CAL27 cells incubated with increasing concentrations of ^89^Zr-DFO-Z_EGFR:03115_ bound vs. ^89^Zr-DFO-Z_EGFR:03115_ incubation concentration. (B) In vitro binding specificity of ^89^Zr-DFO-Z_EGFR:03115_ in selected cell lines with and without blocking using unlabeled Affibody, cetuximab, or EGF. Data are normalized to maximum cell-associated radioactivity per experiment.

The K_d_ and maximum number of binding sites of ^89^Zr-DFO-Z_EGFR:03115_ were determined by a cell-based saturation assay using CAL27 cells. The calculated K_d_ and maximum number of binding sites were 5.00 ± 0.3 nM and (3.71 ± 0.05) × 10^6^ sites per cell, respectively (Fig. 2A). The target-binding specificity of ^89^Zr-DFO-Z_EGFR:03115_ was evaluated in a panel of cancer cell lines with different degrees of EGFR expression, demonstrating that the cell-associated radioactivity was consistent with the measured total EGFR protein expression level in each cell line (Figs. 1A and 2B). Importantly, blocking the receptor with a 100-fold excess of nonlabeled Z_EGFR:03115_, cetuximab, or the natural ligand EGF, which were demonstrated to compete with the Affibody molecule for the same binding site, significantly reduced radioactivity signal, further confirming the specificity of ^89^Zr-DFO-Z_EGFR:03115_ for the target (Fig. 2B).

### Protein Dose-Escalation Studies

**FIGURE 3.**
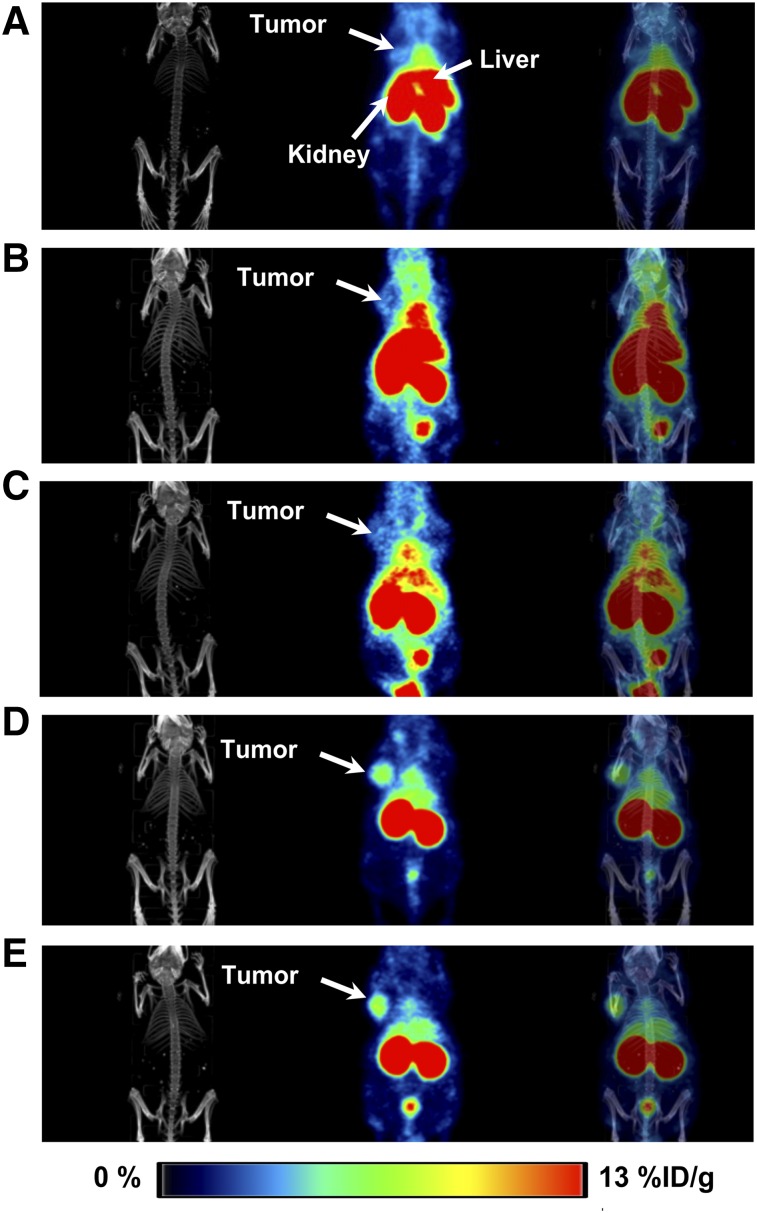
Whole-body coronal PET/CT images acquired 3 h after ^89^Zr-DFO-Z_EGFR:03115_ administration spiked with different amounts of nonlabeled Z_EGFR:03115_ in mice bearing CAL27 tumors: 2 μg of ^89^Zr-DFO-Z_EGFR:03115_ injected (A); 1 μg of Z_EGFR:03115_ injected 30 min before injection of 2 μg of ^89^Zr-DFO-Z_EGFR:03115_ (B); 5 μg of Z_EGFR:03115_ injected 30 min before injection of 2 μg of ^89^Zr-DFO-Z_EGFR:03115_ (C); 10 μg of Z_EGFR:03115_ injected 30 min before injection of 2 μg of ^89^Zr-DFO-Z_EGFR:03115_ (D); 10 μg of Z_EGFR:03115_ coinjected with 2 μg of ^89^Zr-DFO-Z_EGFR:03115_ (E).

Protein dose-escalation studies were initially performed to test the bioavailability of the radioconjugate for tumor targeting. By adding increasing amounts of nonlabeled Affibody molecule (1, 5, 10, 15, and 20 μg) to the ^89^Zr-DFO-Z_EGFR:03115_ (2 μg, 2.5–3.4 MBq/mouse), the radioactivity signal in the liver significantly decreased from 20.74 ± 8.31 to 3.19 ± 0.14 %ID/g (Table 1). Concurrently, tumor uptake increased from 1.75 ± 0.21 to 3.69 ± 1.19 %ID/g (Table 1; Fig. 3). Importantly, an insignificant uptake was achieved when the nonspecific Affibody-based radioconjugate (^89^Zr-DFO-Z_Taq_) was injected in CAL27 tumors (0.26 ± 0.05 %ID/g) (Table 1; Supplemental Fig. B2). Also, no significant differences in tumor uptake (3.69 ± 1.19 %ID/g vs. 3.88 ± 0.46 %ID/g) were found when nonlabeled Z_EGFR:03115_ was either injected 30 min before the radioconjugate or coinjected with the ^89^Zr-DFO-Z_EGFR:03115_ (Supplemental Fig. 2). Therefore, the protein dose of 10 μg was administered alongside the radioconjugate for further studies, as it provided the best contrast between targeted and nontargeted tissues (tumor-to-background ratio of 10.25 at 3 h after injection).

**TABLE 1:** Ex Vivo Biodistribution 3 Hours After Intravenous Administration of Increasing Amounts of Nonlabeled Z_EGFR:03115_ 30 Minutes Before 2 μg of ^89^Zr-DFO-Z_EGFR:03115_ or 2 μg of ^89^Zr-DFO-Z_Taq_ in CAL27 Xenografts

	2 μg ^89^Zr-DFO-Z_EGFR:03115_					
Organ	0 μg Z_EGFR:03115_	1 μg Z_EGFR:03115_	5 μg Z_EGFR:03115_	10 μg Z_EGFR:03115_	20 μg Z_EGFR:03115_	2 μg ^89^Zr-DFO-Z_Taq_ (10 μg Z_EGFR:03115_)
Blood	3.52 ± 1.42	6.01 ± 0.18	3.87 ± 0.30	4.46 ± 1.65	3.06 ± 0.20	0.64 ± 0.04
Heart	0.89 ± 0.22	1.53 ± 0.05	0.95 ± 0.09	1.03 ± 0.29	0.77 ± 0.06	0.25 ± 0.03
Lungs	1.45 ± 0.45	2.55 ± 0.23	1.84 ± 0.50	2.05 ± 0.1.06	1.45 ± 0.08	0.47 ± 0.05
Kidney	37.14 ± 1.17	54.52 ± 17.13	73.26 ± 12.91	140.84 ± 47.70	109.14 ± 22.30	172.04 ± 20.07
Spleen	0.91 ± 0.10	1.58 ± 0.23	0.94 ± 0.20	1.28 ± 0.13	0.94 ± 0.13	0.37 ± 0.08
Liver	20.74 ± 8.31	10.36 ± 1.49	3.82 ± 0.39	4.25 ± 1.55	3.19 ± 0.14	0.82 ± 0.22
Pancreas	0.73 ± 0.19	1.62 ± 0.24	0.71 ± 0.12	0.69 ± 0.13	0.46 ± 0.04	0.14 ± 0.03
Tumor	1.75 ± 0.21	1.70 ± 0.68	1.87 ± 0.58	3.69 ± 1.19	2.59 ± 0.48	0.26 ± 0.05
Bone	1.35 ± 0.24	1.24 ± 0.21	0.81 ± 0.15	0.87 ± 0.14	0.66 ± 0.09	0.21 ± 0.05
Intestine	0.59 ± 0.28	1.62 ± 0.57	0.90 ± 0.11	0.89 ± 0.11	0.81 ± 0.05	0.19 ± 0.03
Muscle	0.24 ± 0.01	0.35 ± 0.07	0.37 ± 0.09	0.36 ± 0.05	0.33 ± 0.04	0.08 ± 0.03
Ratio						
Tumor-to-blood	0.50	0.28	0.48	0.83	0.85	0.41
Tumor-to-muscle	7.29	4.86	5.05	10.25	7.85	3.15
Tumor-to-liver	0.08	0.16	0.49	0.87	0.81	0.32

Data are mean ± SD (*n* ≥ 3) %ID/g.

### ^89^Zr-DFO-Z_EGFR:03115_ Pharmacokinetics

The pharmacokinetics of the ^89^Zr-DFO-Z_EGFR:03115_ were evaluated by performing biodistribution studies at 3, 24, and 48 h after injection of 2 μg (2.5–3.4 MBq) of radioconjugate with 10 μg of Z_EGFR:03115_. The tumor-to-muscle and tumor-to-blood ratios at 3 h were 10.31 and 1.04, respectively. Because of clearance from nonspecific organs, both values increased at 24 and 48 h after injection, with tumor-to-muscle ratios of 17.35 and 13.56, respectively, and tumor-to-blood ratios of 2.79 and 9.34, respectively (Table 2). At the same time, tumor uptake decreased from 3.88 ± 0.46 %ID/g at 3 h to 2.43 ± 0.27 %ID/g and 2.13 ± 0.12 %ID/g at 24 and 48 h, respectively. The higher tumor uptake would allow for better differentiation between the xenograft tumor types, so we therefore decided to perform the subsequent studies at 3 h after injection. As expected, the kidney uptake remained relatively high over time (104.85 ± 11.10 %ID/g even after 48 h) as a result of glomerular filtration of the Affibody molecule followed by reabsorption, degradation, and retention in proximal tubular cells. Additionally, as expected when using ^89^Zr-labeled agents, radioactivity accumulation in the bone at 24 h (2.28 ± 0.27 %ID/g) and 48 h (3.44 ± 0.59 %ID/g) was detected.

**TABLE 2:** Ex Vivo Biodistribution 3, 24, and 48 Hours After Intravenous Injection of 2 μg of ^89^Zr-DFO-Z_EGFR:03115_ Coinjected with 10 μg of Nonlabeled Z_EGFR:03115_ in Mice Bearing CAL27 Xenografts

Organ	3 h	24 h	48 h
Blood	3.70 ± 0.55	0.87 ± 0.32	0.23 ± 0.06
Heart	0.99 ± 0.19	0.37 ± 0.12	0.31 ± 0.04
Lungs	2.74 ± 0.93	0.88 ± 0.13	0.70 ± 0.08
Kidney	130.42 ± 22.25	108.80 ± 19.60	104.85 ± 11.10
Spleen	1.14 ± 0.18	0.72 ± 0.20	0.79 ± 0.37
Liver	4.99 ± 0.85	4.31 ± 1.00	4.11 ± 1.41
Pancreas	0.67 ± 0.14	0.36 ± 0.10	0.35 ± 0.25
Tumor	3.88 ± 0.46	2.43 ± 0.27	2.13 ± 0.12
Bone	0.99 ± 0.28	2.28 ± 0.27	3.44 ± 0.59
Intestine	1.09 ± 0.16	0.42 ± 0.03	0.40 ± 0.02
Muscle	0.38 ± 0.09	0.14 ± 0.04	0.16 ± 0.07
Ratio			
Tumor-to-blood	1.04	2.79	9.34
Tumor-to-muscle	10.31	17.35	13.56
Tumor-to-liver	0.78	0.56	0.52

Data are mean ± SD (*n* ≥ 3) %ID/g.

### Correlation of ^89^Zr-DFO-Z_EGFR:03115_ Tumor Uptake and EGFR Expression

**FIGURE 4.**
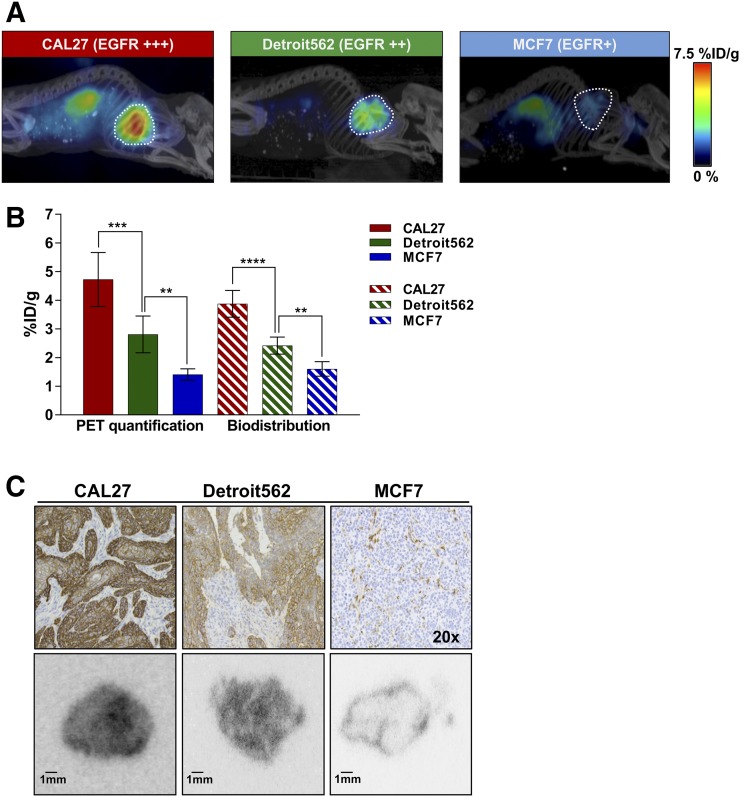
Radioconjugate uptake in xenografts with varying EGFR expression. (A) Representative whole-body sagittal PET/CT images acquired 3 h after injection. (B) PET quantification of radiotracer uptake in tumors (outlined on image) 3 h after injection in comparison with data obtained from biodistribution studies. Data are mean ± SD. (C) Histopathologic analysis of EGFR expression in xenograft models (top), and representative autoradiography tumor sections 3 h after radioconjugate administration (bottom). **P < 0.01. ***P < 0.001. ****P < 0.0001.

To evaluate whether ^89^Zr-DFO-Z_EGFR:03115_ could distinguish between tumors with varying levels of EGFR expression, mice bearing CAL27, Detroit562, and MCF7 xenografts received the radiotracer (2 μg coinjected with 10 μg of Z_EGFR:03115_; 2.4–3.4 MBq/mouse) and were imaged 3 h after injection using PET/CT. The quantified PET imaging data indicated that the highest levels of radioconjugate accumulation were in CAL27 tumors (4.73 ± 0.90 %ID/g) (Figs. 4A and 4B). Importantly, in vivo specificity of Z_EGFR:03115_ was confirmed by the lower ^89^Zr-DFO-Z_EGFR:03115_ uptake in low–EGFR-expressing MCF7 xenografts (1.41 ± 0.20 %ID/g). These data were then corroborated by the corresponding biodistribution results (Fig. 4B). Furthermore, tumor targeting by the radioconjugate correlated with EGFR expression measured ex vivo by Western blot analysis and immunohistochemical staining (Fig. 4C, top; Supplemental Fig. 3). In addition, the autoradiography of tissue slices from CAL27 and Detroit562 tumors confirmed marked differences in ^89^Zr-DFO-Z_EGFR:03115_ uptake as compared with MCF7 tumors (Fig. 4C, bottom).

### Monitoring Cetuximab-Induced In Vivo EGFR Downregulation

**FIGURE 5.**
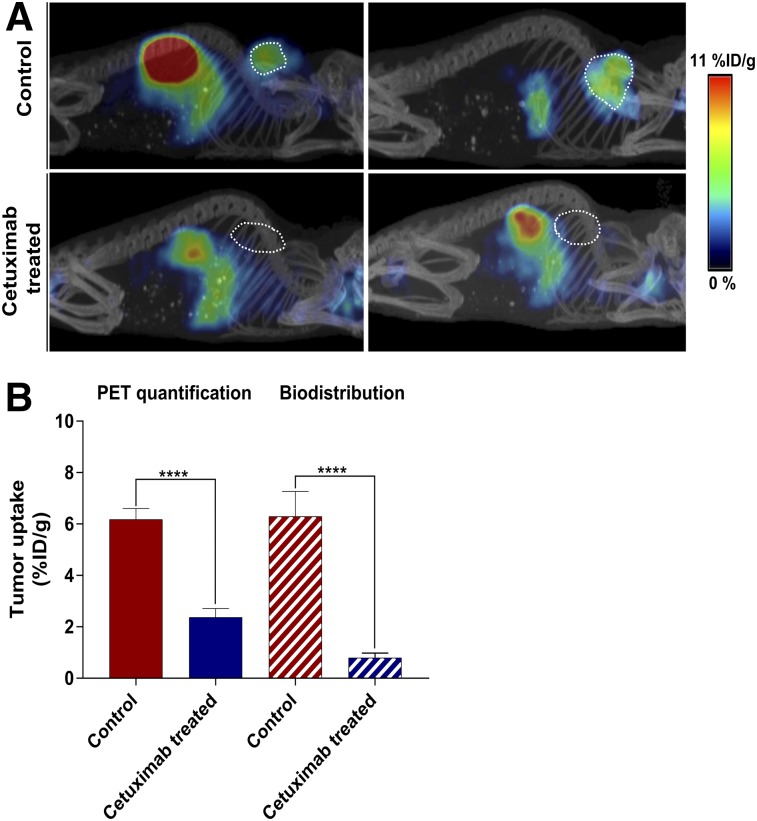
^18^F-AlF-NOTA-Z_EGFR:03115_ uptake assessed 1 h after injection. (A) Representative sagittal whole-body PET/CT images of mice bearing HN5 tumors (outlined on image) with or without treatment with cetuximab. (B) PET quantification in control and cetuximab-treated HN5 tumors and corresponding biodistribution %ID/g values. Data are mean ± SD (*n* ≥ 6). ****P < 0.0001.

**FIGURE 6.**
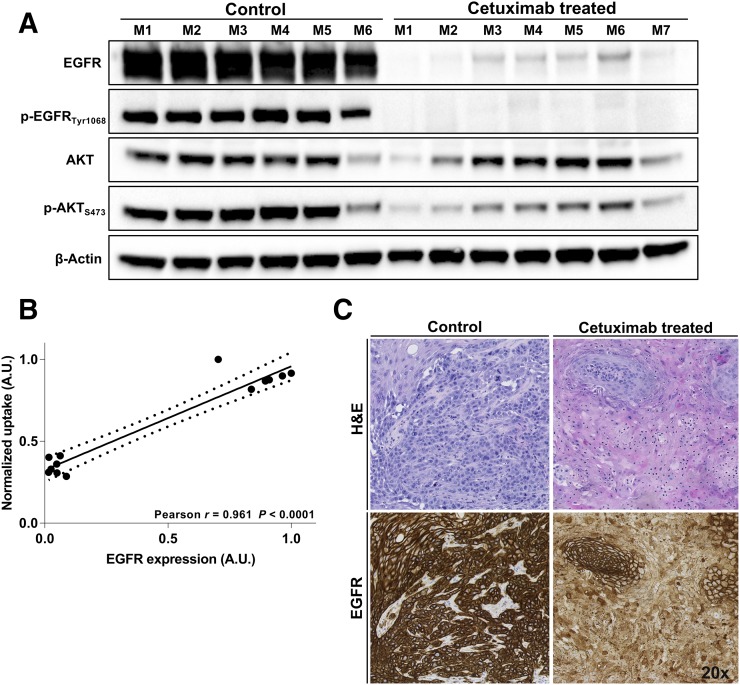
(A) Western blot of tumor tissue lysates from control and cetuximab-treated mice demonstrating EGFR protein expression, activation, and downstream signaling. (B) Spearman rank correlation analysis for EGFR expression as determined by Western blot against ^18^F-AlF-NOTA-Z_EGFR:03115_ tumor uptake as quantified by PET image analysis. Dashed lines represent 95% confidence levels. (C) Histopathologic analysis of EGFR expression and hematoxylin and eosin (H&E) staining in HN5 xenografts in both control and cetuximab-treated mouse. A.U. = arbitrary units.

To monitor the response to cetuximab treatment, the long-lived isotope, ^89^Zr (half-life, 78.4 h), was replaced with ^18^F (half-life, 108 min). In vitro characterization of ^18^F-AlF-NOTA-Z_EGFR:03115_ confirmed the high affinity (K_d_, 5.4 ± 1.1 nM) and specificity of the radioconjugate for EGFR (Supplemental Fig. 4). After ^18^F-AlF-NOTA-Z_EGFR:03115_ intravenous administration to mice bearing HN5 tumors, a significantly lower tumor uptake was observed in cetuximab-treated mice than in control HN5 tumors, as shown both by the PET image quantification (6.29 ± 0.89 %ID/g vs. 2.37 ± 0.31 %ID/g) and by the biodistribution data (6.18 ± 0.38 %ID/g vs. 0.80 ± 0.17 %ID/g) (Fig. 5; Supplemental Fig. 5A). PET quantification of the cetuximab-treated mice yielded a slightly higher %ID/g than the biodistribution studies, probably because of the relatively high radioactivity in the blood at 1 h after injection (Supplemental Figs. 5B and 5C). However, when background subtraction was applied, only a small amount of the activity was present in the treated tumors (0.28 ± 0.26 %ID/g) (Supplemental Fig. 5D). This significant decrease in the radioconjugate accumulation correlated with a downregulation of EGFR expression after cetuximab treatment as assessed by Western blotting (*r* = 0.961, *P* < 0.0001) (Figs. 6A and 6B; Supplemental Figs. 5E and 5F). Furthermore, discernible differences in phosphorylated EGFR and AKT were observed between control and treated mice (Fig. 6A). Additionally, a cetuximab-induced decrease in EGFR expression was confirmed by the immunohistochemistry (Fig. 6C). No significant change in tumor uptake was measured when a group of mice was injected with ^18^F-AlF-NOTA-Z_EGFR:03115_ and a quantity of cetuximab that was estimated to be still circulating in the blood 13 d after treatment (Supplemental Fig. 5F). This finding confirmed that the decrease in tumor uptake was not due to competition of the mAb for the same epitope on the receptor. Additionally, no significant decrease in tumor uptake was measured (Supplemental Fig. 5G).

## DISCUSSION

Despite evidence demonstrating an important prognostic role for EGFR in HNSCC, receptor expression has not been predictive of response to EGFR-targeted therapy. This finding was potentially due to evaluation of EGFR expression in the major clinical trials by an immunohistochemistry-based scoring system, which may not reflect the heterogeneous receptor expression of the whole tumor mass and associated regional lymph node metastases. The integration of molecular imaging biomarkers into standard clinical protocols after injection could address these limitations, providing a global representation of tumor target expression and accessibility, enabling image-guided selection of patients for EGFR-targeted therapies.

Recently, therapeutic mAbs targeting EGFR have been radiolabeled with multiple radioisotopes to provide information about tumor targeting, pharmacokinetics, and accumulation in critical normal organs (23–25). Even et al. reported that ^89^Zr-cetuximab PET shows large interpatient variability in locally advanced HNSCCs and provides additional information about the accessibility of the drug into the tumor when compared with ^18^F-FDG PET and EGFR expression evaluated by ex vivo methods (26). However, Niu et al. have demonstrated in different HNSCC xenografts that poor ^64^Cu-DOTA-panitumumab delivery may result in lack of correlation between PET quantification and the EGFR protein expression (27).

We therefore used a low-molecular-weight targeting vector (i.e., Affibody molecule), because the rapid extravasation from the blood vessels and enhanced tumor mass penetration would provide favorable pharmacokinetics for imaging applications and facilitate visualization of tumor regions inaccessible to mAbs, particularly at early time points. In fact, van Dijk et al. have shown high and specific uptake of ^111^In-cetuximab-F(ab′)_2_ as early as 4 h after injection, with high imaging contrast at 24 h in FaDu xenografts. The authors additionally reported that the radioconjugate can monitor the effects of EGFR inhibition combined with irradiation in head and neck carcinoma models (23,28). However, EGFR-targeting mAb fragments do not bind to murine EGFR, limiting proper evaluation of the real tumor-to-background signal of these agents in preclinical models. To address this limitation, several recent studies have investigated whether EGFR-specific Affibody molecules, which cross-react with EGFR of mouse origin, can delineate EGFR-positive tumors and provide high-contrast tumor imaging in the presence of endogenous background levels of EGFR (15–17). In our study, we investigated the binding of Z_EGFR:03115_ in a panel of HNSCC models. The in vitro data clearly demonstrated that the fluorescent (Z_EGFR:03115_-Dylight633) and radiolabeled (^89^Zr-DFO-Z_EGFR:03115_, ^18^F-AlF-NOTA-Z_EGFR:03115_) Affibody-based conjugates maintained a high binding affinity for EGFR and enabled detection of differences in EGFR expression. The specificity for EGFR binding was confirmed by blocking assays with cetuximab and the natural ligand, EGF. To study the pharmacokinetics of Z_EGFR:03115_ at later time points, we radiolabeled the Affibody molecule with a long-lived PET radioisotope (17). ^89^Zr was seen as a better-suited radionuclide than ^124^I for such studies because radiometals are retained more readily than radiohalogens in cells.

Numerous studies have highlighted the importance of optimizing the protein dose when targeting EGFR in order to partially saturate endogenous EGFR expression in the liver and, subsequently, increase the tumor uptake (29,30). Therefore, we performed a protein dose-escalation study and found that we can clearly visualize the tumor 3 h after coinjecting 10 μg of Z_EGFR:03115_ alongside ^89^Zr-DFO-Z_EGFR:03115_ (tumor-to-muscle ratio of 10.31). Furthermore, radioconjugate tumor accumulation was confirmed to be EGFR-specific, as PET imaging of MCF-7 (EGFR+) showed much lower accumulation of the radioconjugate than tumors with higher receptor expression. Moreover, the ^89^Zr-DFO-Z_Taq_–related radioactivity in the CAL27 tumor (EGFR++++) was negligible. Importantly, the accumulation of ^89^Zr-DFO-Z_EGFR:03115_ in the tumor correlated with EGFR protein expression level, assessed ex vivo by tumor tissue lysates and EGFR staining of tumor sections derived from xenograft models with various levels of receptor expression. Our results successfully demonstrated the advantage of performing imaging at later time points with the Affibody molecule, as exemplified by the increase in tumor-to-background ratios. However, the decrease in tumor accumulation after 3 h after injection would limit sensitivity for discerning subtle changes in EGFR expression. Therefore, the 3-h time point was selected to compare the receptor levels between the xenograft models. Furthermore, the clinical use of an Affibody conjugate radiolabeled with ^18^F would be more suitable than that of an Affibody conjugate radiolabeled with ^89^Zr because of a lower radiation exposure burden to the patient, a more desirable positron branching ratio, and a shorter radioactive half-life (108 min vs. 78.4 h). We postulated that ^18^F-AlF-NOTA-Z_EGFR:03115_ could measure dynamic changes in receptor expression in response to EGFR inhibition.

This ability may provide useful information for adaptive treatment schedules with anti-EGFR mAbs, since current dosing of cetuximab is based on the patient’s weight and the only confirmed clinical variable that predicts response to this agent is the grade of skin toxicity that occurs after treatment initiation (31,32). Notably, after cetuximab treatment, we observed a significantly lower radioconjugate uptake in the group of mice treated with this mAb. Moreover, this change was in line with decreased EGFR protein levels evaluated by Western blot and immunohistochemistry. These results, together with the fact that no significant change in tumor volume was observed during the treatment, highlight the potential for using EGFR imaging as a tool for assessing cetuximab efficacy based on the receptor level rather than relying purely on anatomic imaging. Of note, EGFR downregulation, because of the internalization and subsequent degradation of EGFR in lysosomes, has recently been reported to be an important determinant of the efficacy of cetuximab treatment for colorectal cancer (33). Our data suggest that it may be possible to evaluate this effect noninvasively using PET-based imaging with radiolabeled Affibody molecules.

Furthermore, immunostaining of tumor sections revealed the presence of residual EGFR-positive cells that directly reflected their viability after cetuximab treatment. These results were corroborated by incomplete inhibition of p-AKT signaling. The surviving cell populations were likely inaccessible to the bulky antibody but potentially may have evaded cetuximab treatment by activating compensatory signaling pathways. However, we recognize that further investigations are needed to verify these findings, which may point the way to further treatment options for such tumors. In the future, ^18^F-AlF-NOTA-Z_EGFR:03115_ could also be used for the identification of residual EGFR-positive cells that may be responsible for subsequent cancer relapse.

## CONCLUSION

Our results demonstrate that noninvasive molecular imaging of EGFR may open the door to guiding the selection and monitoring of anti-EGFR targeted therapy before it is possible to detect tumor response by changes in volume on conventional cross-sectional imaging modalities. Such an approach may spare patients unnecessary toxicities and improve EGFR-targeted therapy by tailoring a more personalized treatment.

## DISCLOSURE

This research was supported in part by the Cancer Research U.K. Cancer Imaging Centre (C1060/A16464) and EPSRC (EP/H046526/1). This report is independent research funded by the National Institute for Health Research. The views expressed in this publication are those of the authors and not necessarily those of the NHS, the National Institute for Health Research, or the Department of Health. No other potential conflict of interest relevant to this article was reported.
