# Supplementary material for: Affibody-Based PET Imaging to Guide EGFR-Targeted Cancer Therapy in Head and Neck Squamous Cell Cancer Models
Source: J Nucl Med. 2019 Mar;60(3):353–61. doi: 10.2967/jnumed.118.216069 (PMC6424230; doi:10.2967/jnumed.118.216069)
Supplement: Supplementary file 1 [file jnm216069SupplementalData.pdf]

Part A : Preparation of conjugates and stability of radioconjugates

Radiosynthesis materials

All the reagents and solvents were purchased from commercial sources and used without further purification unless otherwise stated. The $Z_{EGFR:03115}$-Cys And Z_{Taq} Affibody molecules was provided by Affibody AB (Solna, Sweden http://www.affibody.com) as a solution in 0.2M sodium acetate pH 5.3. HPLC grade acetonitrile, water, trifluoroacetic acid (TFA), tris(2-carboxyethyl)phosphine hydrochloride (TCEP-HCl), and sodium carbonate (Na_2CO_3) were purchased from ThermoFisher Scientific (Loughborough, UK). Ethylenediaminetetraacetic acid (EDTA), sodium acetate, dimethyl sulfoxide (DMSO,) sodium citrate tribasic, and mouse serum were purchased from Sigma Aldrich (Gillingham, Dorset, UK). Phosphate buffered saline (PBS) was purchased from Gibco (Life Technologies, Paisley, UK). Deferoxamine-maleimide (DFO-maleimide) was purchased from Macrocyclics, US. Maleimidoethylmonoamide NOTA (MMA-NOTA) was purchased from Chematech, France. Aluminum chloride hexahydrate ($AlCl_3$, 99.9995%) was purchased from Alfa Aesar (Heysham, UK). Low-protein binding microcentrifuge tubes (1.5 mL) were purchased from Eppendorf (Sigma Aldrich, UK). Silica-glass (SG) ITLC chromatography paper was purchased from Agilent Technologies. Salicylic acid (SA) ITLC chromatography paper used for serum stability analysis was generously gifted by Agilent Technologies. ^{89}Zr-oxalate was purchased from BV Cyclotron VU (Perkin Elmer, Amsterdam, NL). ^{18}F-Fluoride was produced a GE PETrace cyclotron by 16 MeV irradiation of an enriched [^{18}O]H_2O target, supplied by Alliance Medical Radiopharmarcy Ltd (Warwick, UK) and used without further purification. Lyophilization was performed on a Concentrator Plus (Eppendorf, Stevenage, UK). Sample incubation was performed using a Thermomixer (Eppendorf, Stevenage, UK). UV absorbance was measured on a Nanodrop 2000 (ThermoFisher Scientific, UK). Oasis HLB 1 mL 30 mg SPE cartridges were purchased from Waters (Elstree, UK). The synthesis and radiolabeling with ^{89}Zr of DFO-Z_{Taq} was performed as described in the literature .

Analytical and semi-prep RP-HPLC were carried out on an Agilent Infinity 1260 quaternary pump system equipped with a 1260 Diode array (Agilent Technologies). Elution profiles were analysed using Laura software (Lablogic, UK). The radioactivity of the eluate was monitored using an IN/US Systems Gamma-ram Model 4 NaI radiodetector (Lablogic, Sheffield, UK). Retention times (R_t) are expressed as minutes:seconds (min:sec).The radiochemical conversion (RCC) was calculated as the percentage of the ratio between the integral of the peak corresponding to the radioconjugate and the integral of the whole run. Purification of DFO-$Z_{EGFR:03115}$ and NOTA-$Z_{EGFR:03115}$ was performed by semi-Prep HPLC on a Jupiter 10µm C18 300A, 250×10mm column (Phenomenex, UK) using **Gradient 1**: 0 min=30%B, 20 min = 65% B, 22 min = 30% B with aqueous 0.1% TFA and 0.1% TFA in Acetonitrile as eluent A and B respectively. Flow rate = 3 mL/min. Purification of ^{18}F-AlF-NOTA-$Z_{EGFR:03115}$ was performed on a Zorbax 300SB-C18 5µm, 250×4.6mm column (Agilent, UK) using **Gradient 3**: 0 min=35%B; 15 min = 60% B, 16 min = 35% B with aqueous 0.1% TFA and 0.1% TFA in Acetonitrile as eluent A and B respectively. Flow rate = 1 mL/min.

Analytical HPLC samples were analyzed on a Zorbax 300SB-C18 5µm, 250×4.6mm column (Agilent, UK) using **Gradient 2**: 0 min=30%B, 20 min = 65% B, 22 min = 30% B with aqueous 0.1% TFA and 0.1% TFA in Acetonitrile as eluent A and B respectively. Flow rate = 1 mL/min.

SDS-PAGE gels were analyzed using a Typhoon FLA 7000 scanner (GE Healthcare, Chicago, IL) and silver stain kit from Pierce, Thermo Fisher Scientific, UK.

Synthesis of DyLight633-$Z_{EGFR:03115}$

$Z_{EGFR:03115}$-Cys (125 µg, 18.6 nmol) and a 90-fold molar excess of TCEP-HCl (478 µg, 1.67 µmol), were heated for 5 min at 85°C followed by 25 min at RT . The reaction mixture was run through a Zeba Spin Desalting Column (7K MWCO, 2 mL) before the addition of an excess of DyLight633-maleimide (202 µg, 186 nmol). After a 2 h incubation at 40°C, the conjugate was purified through a Zeba Spin Desalting Column (7K MWCO, 5 mL). The protein concentration measured by determining the absorbance at 280nm and divided by the absorption coefficient provided by Affibody (1 Abs_{280} = 0.405 mg/ml). Confirmation of the DyLight633 conjugation of $Z_{EGFR:03115}$ was performed by Tricine SDS-PAGE using fluorescence and silver staining to visualize the product band.

Synthesis and characterization of DFO-Z$_{EGFR:03115}$

<u>Conjugation of DFO to Z$_{EGFR:03115}$</u>

In a 1.5 mL low-protein binding centrifuge tube, a 2.3 mg/mL solution of Z$_{EGFR:03115}$-Cys in 0.2 M sodium acetate (175 µL, ca 59.8 nmol) was diluted with PBS (150 µL) and 0.1 M EDTA (40 µL). A freshly prepared 0.05 mg/µL TCEP-HCl in PBS solution (8.6 µL, 1.50 µmol) was added, and the mixture was incubated in a thermomixer at 85°C for 5 min (850 rpm) followed by 25 min at room temperature. A 0.024mg/mL solution of DFO-maleimide in DMSO was then added (70.8 µL, 1.7 mg, 2.39 µmol) and the solution was incubated in a thermomixer at 37 °C for 2 h (850 rpm). The product was purified by semipreparative reverse phase high performance liquid chromatography (RP-HPLC) using Gradient 1. The fractions containing the product were lyophilized and quantified by measuring the UV absorbance at 280 nm and using the coefficient 1 Abs$_{280}$ = 0.405 mg/mL (278 µg, 49.0% yield). Analytical RP-HPLC was performed using Gradient 2: R$_t$ = 10:51 min:sec

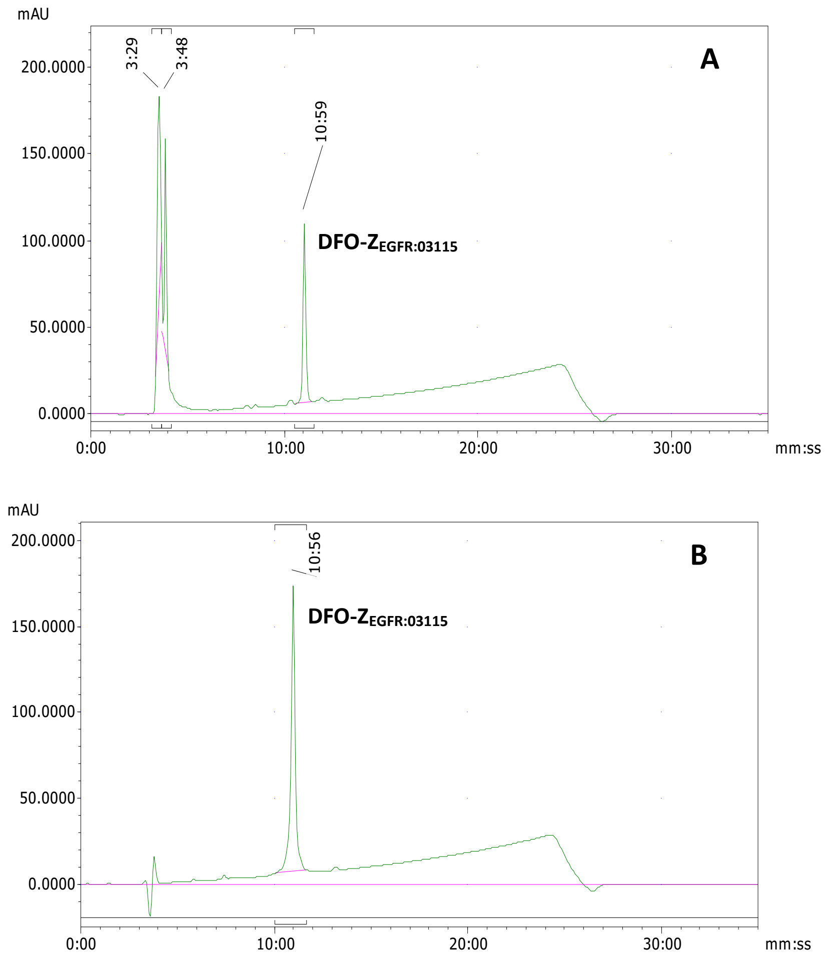

Radiosynthesis Fig. 1. HPLC chromatograms of (**A**) DFO-Z$_{EGFR:03115}$ reaction mixture and (**B**) purified DFO-Z$_{EGFR:03115}$ analyzed using Gradient 2. The absorbance was recorded at the wavelength of 230 nm. The excess of TCEP and DFO-maleimide elutes at the solvent front.

MALDI-MS analysis of DFO-$Z_{EGFR:03115}$

Matrix-assisted laser desorption ionization mass spectrometry (MALDI-MS) analyses were performed by the EPSRC UK National Mass Spectrometry Facility at Swansea University, UK
MALDI-MS (m/z): $[M + H]^+$ expected: 7439, found: 7439; $[M + Na]^+$ 7462.

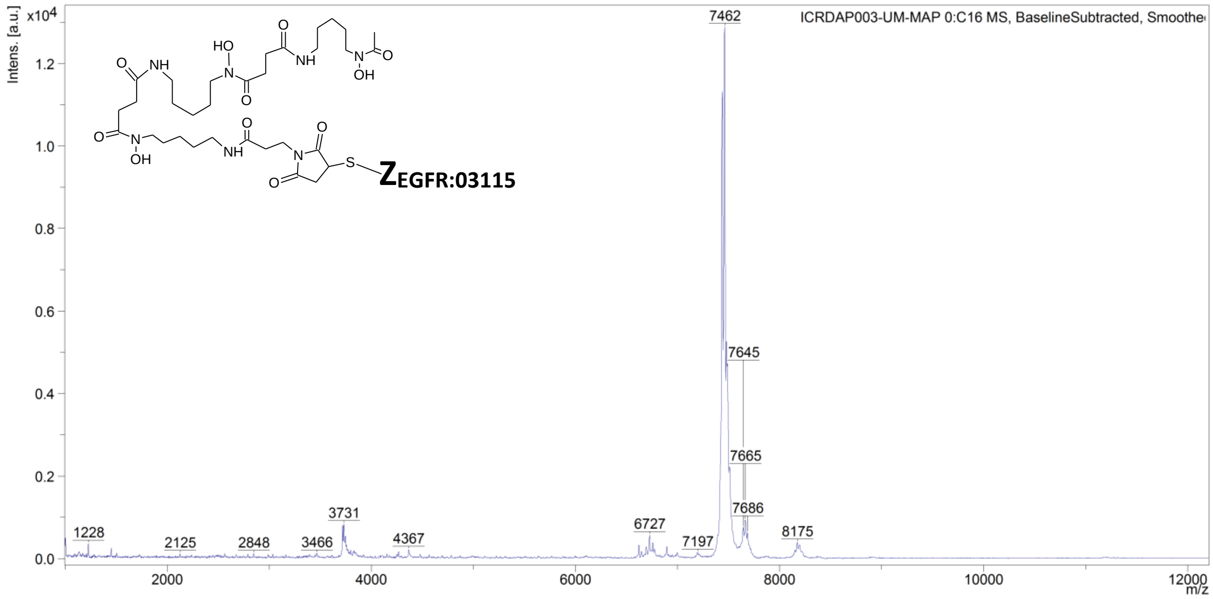

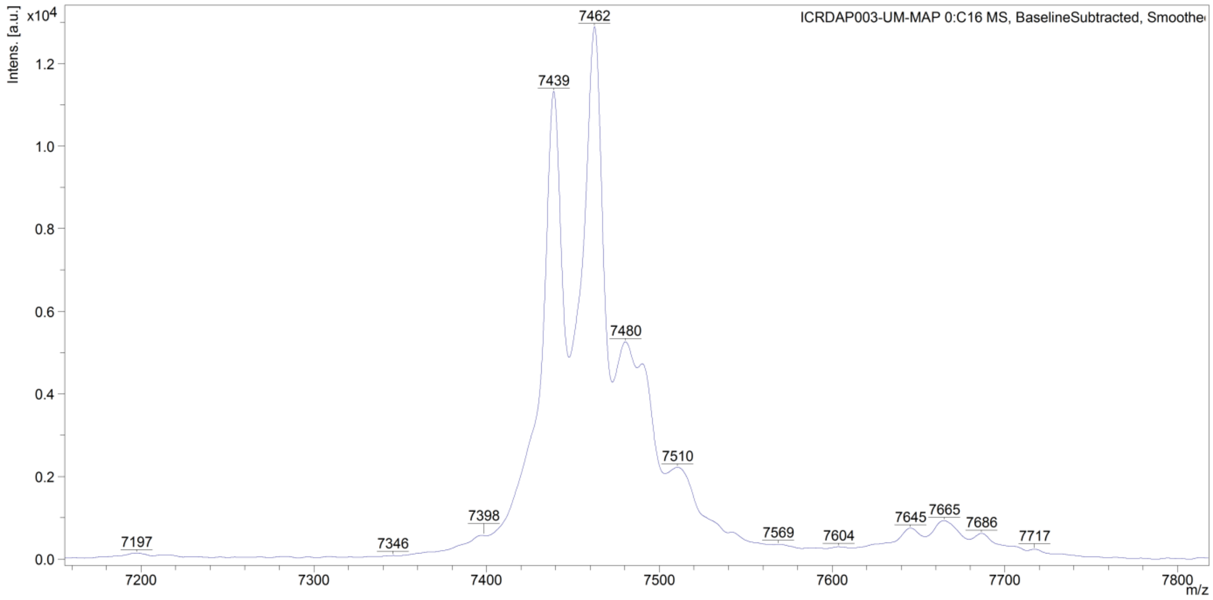

Preparation and *in vitro* serum stability of ^{89}Zr-DFO-Z$_{EGFR:03115}$

Preparation of ^{89}Zr-DFO-Z$_{EGFR:03115}$

The radiolabeling was performed following a published procedure . To optimize the radiolabeling efficiency (>95%), several small reactions (containing max 18 MBq of buffered ^{89}Zr solution) were performed in preference of a large single reaction. In short, the required amount of ^{89}Zr-oxalic acid solution (ca 54-72 MBq) was transferred into a 1.5 mL centrifuge tube followed by a freshly prepared 2.0 M Na_2CO_3 solution in a ^{89}Zr to Na_2CO_3 ratio of 1:0.45 (v/v). The mixture was incubated for 3 min at room temperature. Successively, 0.5 M HEPES (pH 7.1) was added to the solution in a HEPES to ^{89}Zr solution ratio of 2.5:1 (v/v) to have a final pH of 7. The DFO-conjugate in HEPES pH 7.1 (1 mg/mL, 11.00-12.00 µL) was added to aliquots of the buffered ^{89}Zr solution (18 MBq). The mixture was incubated for 1 h at room temperature. The radiolabeling efficiency was determined by ITLC using ITLC-SG strips and 0.1 M citrate buffer (pH 5.0) as eluent. The radioconjugate appears at the origin (R_f = 0.0-0.1) while the $^{89}Zr^{4+}$ which is not bound to the affibody molecule is running with the solvent front (R_f = 0.9-1.0).

Radiometal incorporation ≥ 95%. Final specific activity 1.5-1.6 MBq/µg.

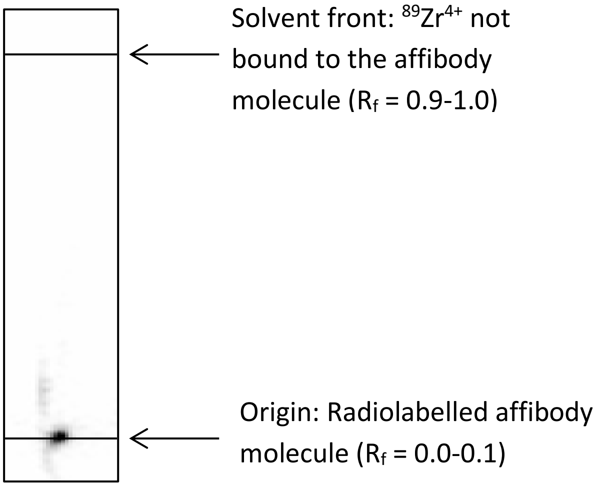

Radiosynthesis Fig. 2. Representative radio-ITLC of the crude radiolabeling mixture of ^{89}Zr-DFO-Z$_{EGFR:03115}$. Radio-ITLC was run on silica-impregnated ITLC strips using 0.1 M citrate buffer (pH 5.0) as the mobile phase.

In vitro serum stability of ^{89}Zr-DFO-Z$_{EGFR:03115}$

The stability of ^{89}Zr-DFO-Z$_{EGFR:03115}$ with respect to loss of radioactivity from the radiolabeled affibody molecule, was assessed by incubating the radioconjugate (ca 1 MBq) in mouse serum (500 µL) in a Thermomixer at 37°C for 1 h (300 rpm). Samples were taken at 1 and 3 h and analyzed by ITLC-SG using 0.1 M citrate buffer (pH 5.0) as eluent. ^{89}Zr-oxalate solution (ca 1 MBq) incubated in mouse serum (500 µL) was processed in the same way and used as control. The experiments were performed in triplicate. The data are expressed as the average of n = 3 measurements ± SD (ITLC image). RCP_{serum} = 94.6±0.5%

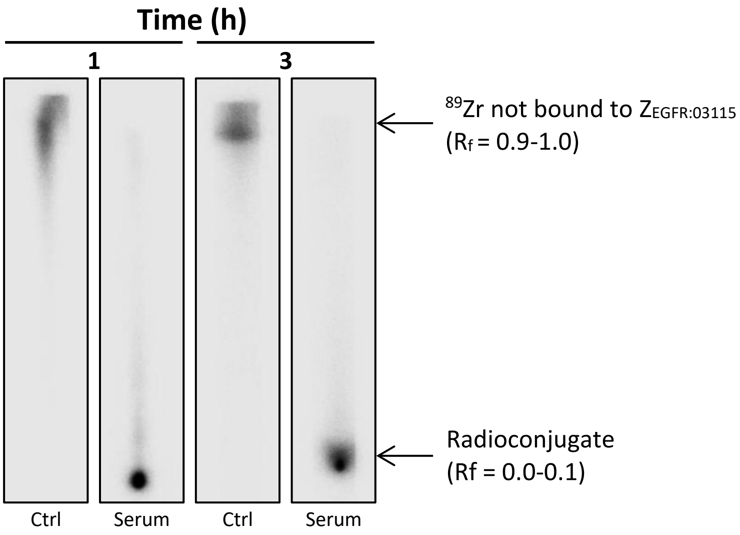

Radiosynthesis Fig. 3. Representative radio-ITLC of ^{89}Zr-DFO-Z$_{EGFR:03115}$ after incubating in mouse serum for 1 and 3 h. Radio-ITLC was run on silica-impregnated ITLC strips using 0.1 M citrate buffer (pH 5.0) as the mobile phase. The ^{89}Zr-oxalate solution in mouse serum was used as control (Ctrl).

Synthesis and characterization of NOTA-Z$_{EGFR:03115}$

Conjugation of NOTA to Z$_{EGFR:03115}$

In a 1.5 mL low protein binding centrifuge tube, a 2.3 mg/mL solution of Z$_{EGFR:03115}$-Cys in 0.2 M sodium acetate (300 µL, ca 102 nmol) was diluted with PBS (250 µL) and 0.1 M EDTA (60 µL). A freshly prepared 0.05 mg/µL TCEP-HCl in PBS solution (14.6 µL, 2.55 µmol) was added, and the mixture was incubated in a thermomixer at 85°C for 5 min (850 rpm) followed by 25 min at room temperature. Maleimidoethylmonoamide NOTA (MMA-NOTA) was then added (2.2 mg, 4.0 µmol) to the solution which was incubated in a thermomixer at 37 °C for 2 h (850 rpm). The product was purified by semipreparative reverse phase high performance liquid chromatography (Gradient 1). The fractions containing the product were lyophilized and quantified by measuring the UV absorbance at 280 nm on a Nanodrop 2000 using the coefficient 1 Abs_{280} = 0.405 mg/mL (387.6 µg, 53.2% yield). Analytical RP-HPLC was performed using Gradient 2: R_t = 10:51 min:sec

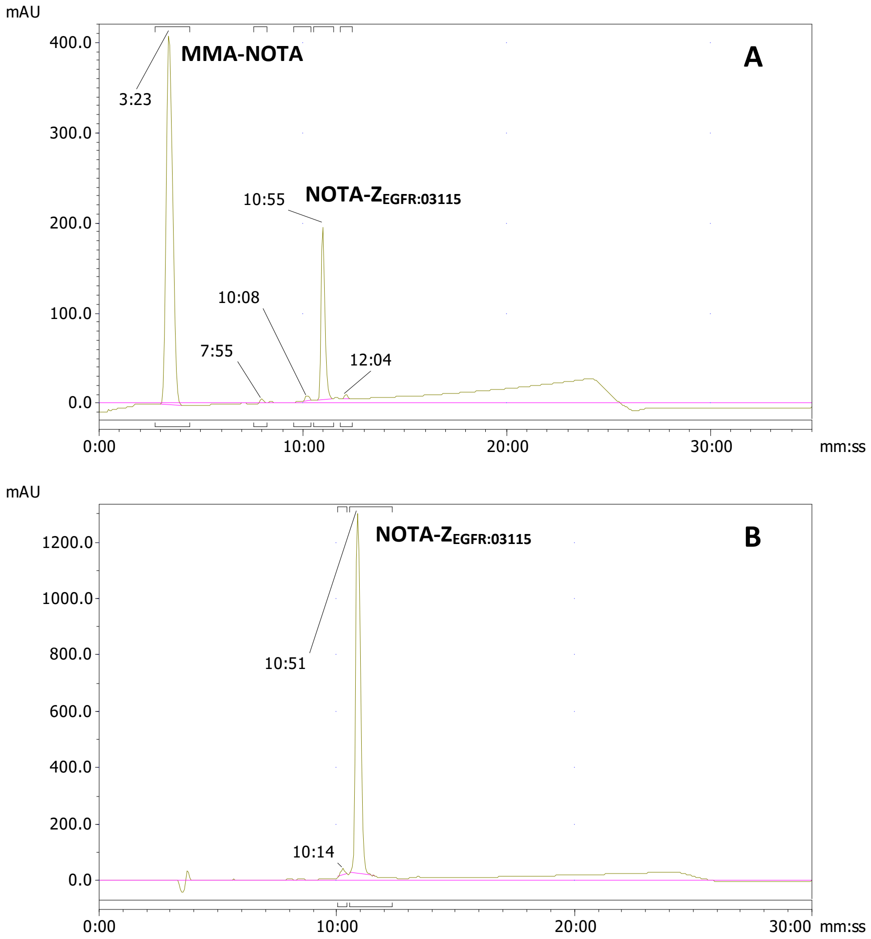

Radiosynthesis Fig. 4. HPLC chromatograms of (**A**) NOTA-$Z_{EGFR:03115}$ reaction mixture and (**B**) purified NOTA-$Z_{EGFR:03115}$ analyzed using Gradient 2. The absorbance was recorded at the wavelength of 230 nm. The excess of MMA-NOTA elutes at 3:23 min:sec.

MALDI-MS analysis of NOTA-$Z_{EGFR:03115}$

Matrix-assisted laser desorption ionization mass spectrometry (MALDI-MS) analyses were performed by the EPSRC UK National Mass Spectrometry Facility at Swansea University, UK

MALDI-MS (m/z): $[M + H]^+$ expected: 7152, found: 7152.

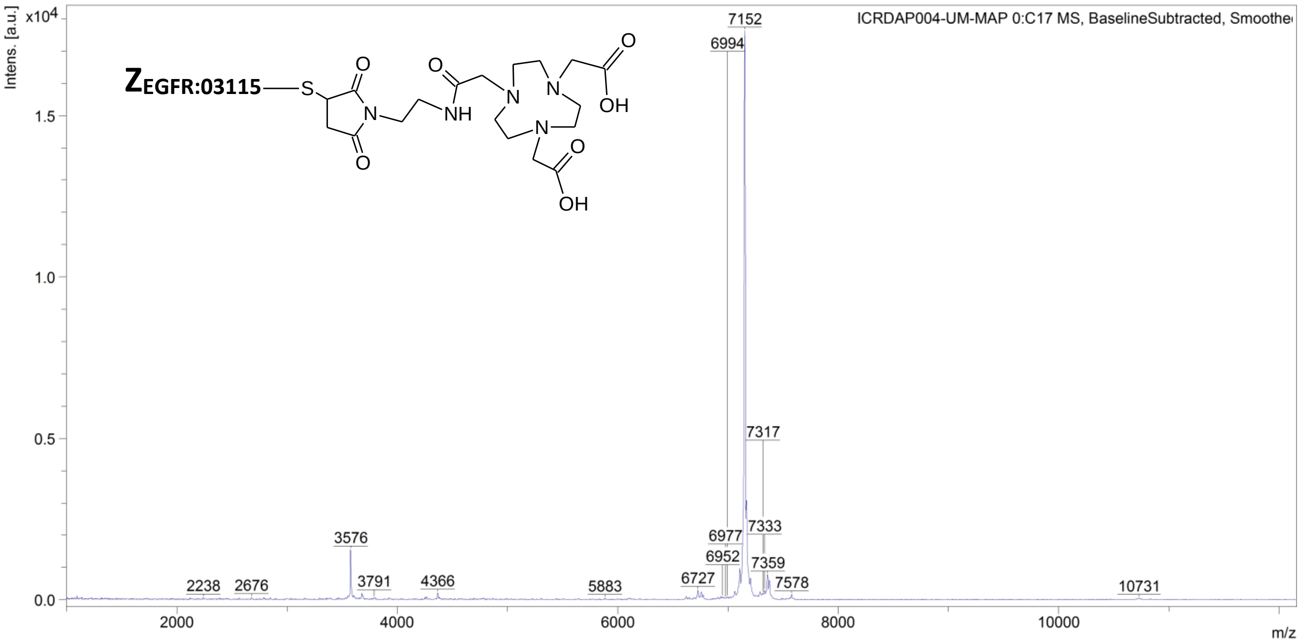

Preparation and *in vitro serum* stability of ^{18}F-AlF-NOTA-Z$_{EGFR:03115}$

Preparation of ^{18}F-AlF-NOTA-Z$_{EGFR:03115}$

To a 1.5 mL low protein binding plastic tube containing the lyophilized NOTA-Z$_{EGFR:03115}$ (15 nmol), 2 mM AlCl3 (6.0 µL, 12 nmol) in 0.5 M sodium acetate buffer at pH 4, aqueous non-purified ^{18}F-fluoride (150-200 MBq), followed by an equal volume of ethanol, were added. The mixture was heated at 100°C for 15 min. After cooling to ambient temperature, the solution was diluted with 0.1% aq TFA (70 µL) and purified by RP-HPLC using Gradient 3: R_t = 8:30 (min:sec). The collected fraction containing the product was diluted with 0.1% aq TFA (3 mL) and loaded on an Oasis HLB-SPE cartridge (1 mL, 30 mg). The trapped radioactivity was washed with 0.1% aq TFA (4 mL) and then eluted with 60% ethanol/water (v/v, ca 120 µL). The product was quantified by measuring the UV absorbance at 280 nm on a Nanodrop 2000 using the coefficient 1 Abs280 = 0.405 mg/mL. Synthesis time (from the beginning of the reaction) = ca. 40 min. Specific activity (decay corrected to end of reaction-purification) = 0.57-1.09 MBq/µg (4.15-7.84 MBq/nmol). Protein recovery = 10.56−34.5%; RCY (decay corrected) = 10.7−38.0%

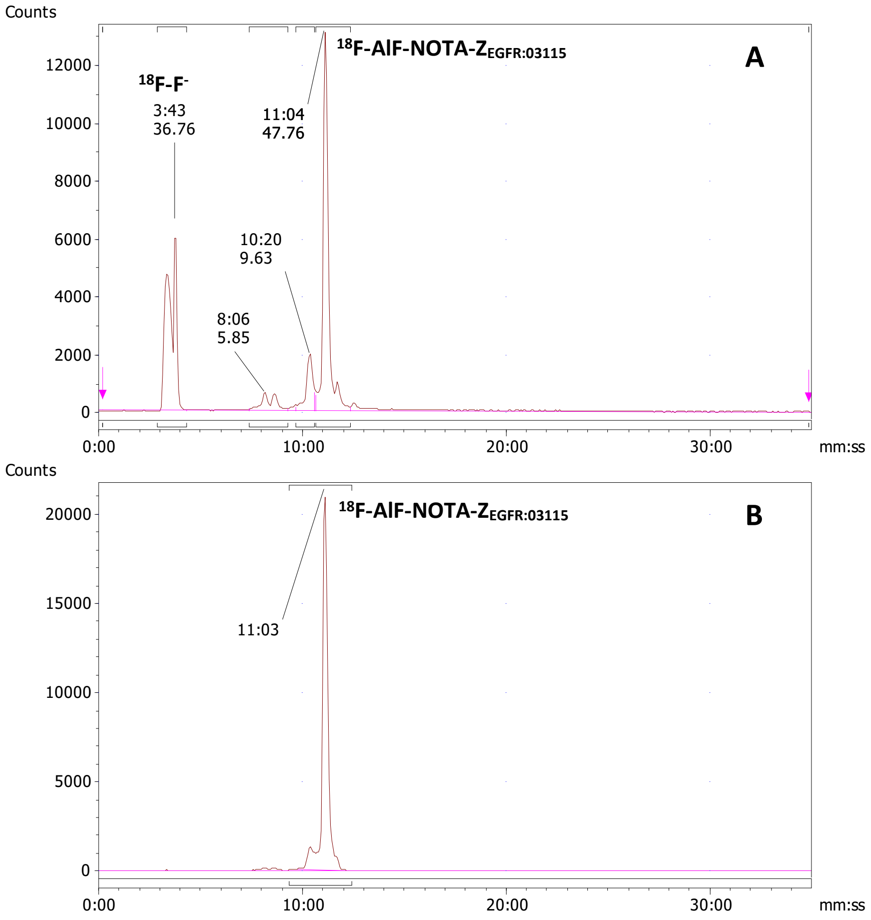

Radiosynthesis Fig. 5. HPLC radiochromatograms (Gradient 2) of: (**A**) ^{18}F-AlF-NODA-Z$_{EGFR:03115}$ reaction mixture. The excess of ^{18}F elutes at the solvent front (R_t = 3:43 min:sec); (**B**) the purified radioconjugate eluting at 11:03 min:sec

Determination of the distribution coefficient (LogD) at pH 7.4 of ^{18}F-AlF-NOTA-$Z_{EGFR:03115}$

^{18}F-AlF-NODA-$Z_{EGFR:03115}$ (ca 0.05MBq) was added to 0.5 mL of PBS (pH 7.4). An equal volume of n-octanol was added and the mixture was vortexed for 10 min followed by centrifugation at 100 × g for 10 min. The experiments were performed in triplicate. Three 100 µl samples were taken from each layer and the amount of activity was measured in a 2480 WIZARD^2 Automatic Gamma Counter (Perkin Elmer, UK) as counts per minutes (cpm). The distribution coefficient at pH 7.4 ($logD_{7.4}$) was expressed as the mean ± standard deviation (SD) and calculated using the formula:

LogD = log[(countsoctanol)/(countsPBS)]

Result: $LogD_{7.4}$ = - 1.13 ± 0.1

In vitro serum stability of ^{18}F-AlF-NOTA-$Z_{EGFR:03115}$

The stability of ^{18}F-AlF-NOTA-$Z_{EGFR:03115}$ with respect to change in RCP and loss of radioactivity from the affibody molecule, was assessed by incubating the purified radioconjugate (ca 4.5 MBq) in mouse serum (500µL, Sigma-Aldrich, UK) in a thermomixer at 37 °C for 1 h (850 rpm). Ethanol (300 µL) was added and the suspension was centrifuged at 16000 × g for 2 min at 22 °C. The supernatant was separated from the pellet and DMF (300 µL) was added to the supernatant. The resulting suspension was centrifuged at 16000 × g for 2 min at 22 °C. The supernatant was separated from the pellet, acidified with 0.1% aq TFA (300 µL), filtered through a 0.2 µ Iso-Disc PVDF syringe filter, and analyzed by RP-HPLC using Gradient 2. The radioactivity associated with the pelleted proteins was measured in a dose calibrator. Aqueous non-purified ^{18}F-Fluoride solution (ca. 4 MBq) incubated in mouse serum (500 µL) was processed in the same way and used as control to confirm the R_t of free ^{18}F-Fluoride. The quantity (%) of intact radioconjugate was calculated as the ratio between the integral of the peak corresponding to the radioligand and the integral of the whole run. The data are expressed as the average of n = 3 measurements ±SD. RCP_{serum} = 95.82 ± 0.67%.

A residual activity associated with the pelleted protein (30.49 ± 2.1%) shows some nonspecific affinity of the radioconjugate toward the serum proteins.

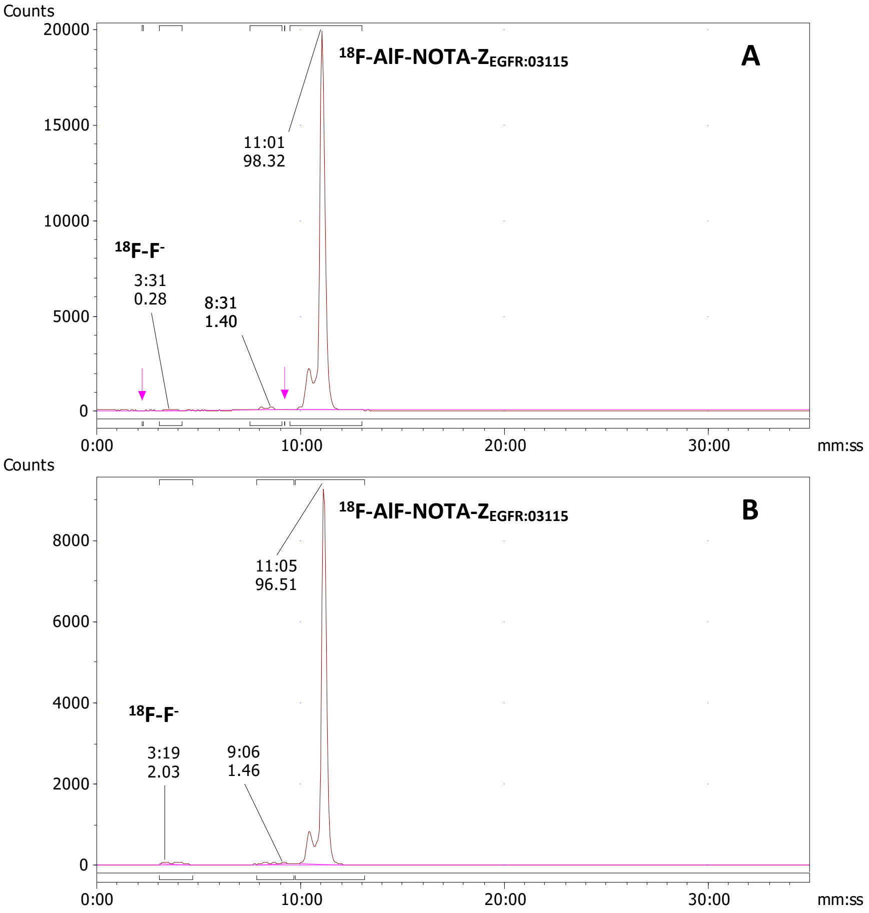

Radiosynthesis Fig. 6: Representative radiochromatograms of purified ^{18}F-AlF-NOTA-Z$_{EGFR:03115}$. (**A**) at time 0 min, and (**B**) after incubation in mouse serum for 1 h using Gradient 2. The intact radioconjugate elutes at ca 11 min. Free ^{18}F-Fluoride elutes at ca 3 min. Labels on each peak on the chromatograms indicate the retention time (top) and the %ROI (bottom).

Part B: *In vitro* cell studies and *in vivo* work

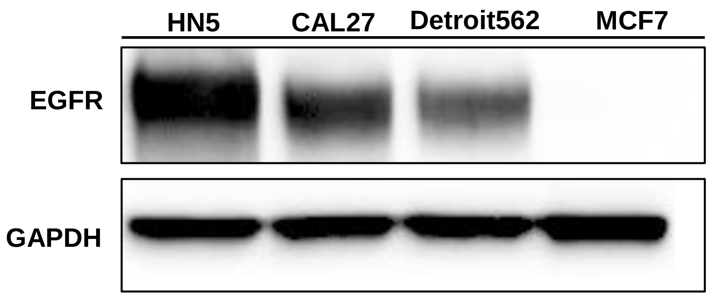

Supplemental Fig. 1: Western blot of whole cell lysates showing the varying EGFR expression in the selected cancer cell lines. GAPDH was used as a loading control.

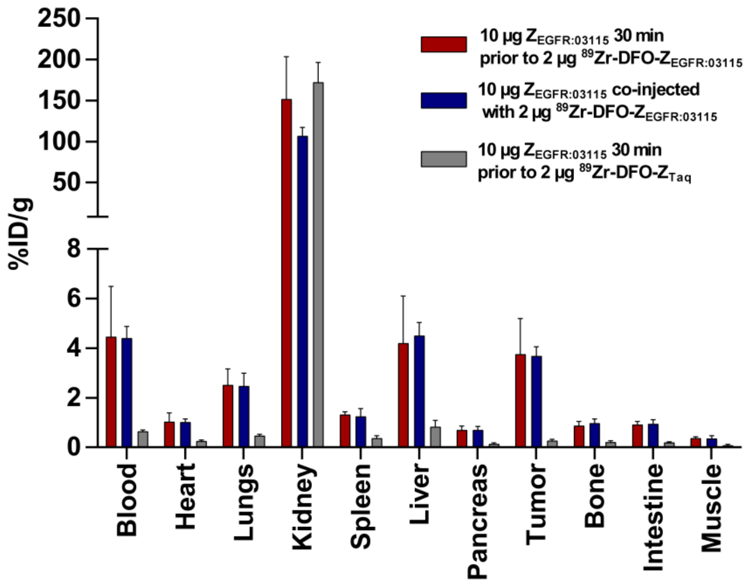

Supplemental Fig. 2: *Ex vivo* biodistribution at 3 h after tail vein injection of ^{89}Zr-DFO-$Z_{EGFR:03115}$ or non-specific ^{89}Zr-DFO-Z_{Taq} in mice bearing CAL27 tumors. The quantity of non-radiolabeled $Z_{EGFR:03115}$ was either co-injected or pre-injected with ^{89}Zr-DFO-$Z_{EGFR:03115}$. Data is reported as mean ± SD (n = 3-7) of injected dose per gram of tissue.

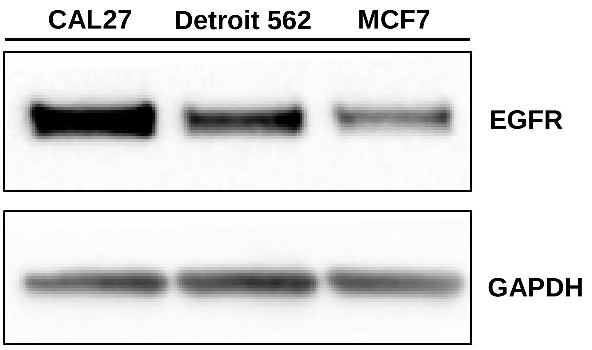

Supplemental Fig. 3: Representative Western blot of tumor tissue lysates to evaluate EGFR expression of the xenografts. GAPDH was used as a loading control.

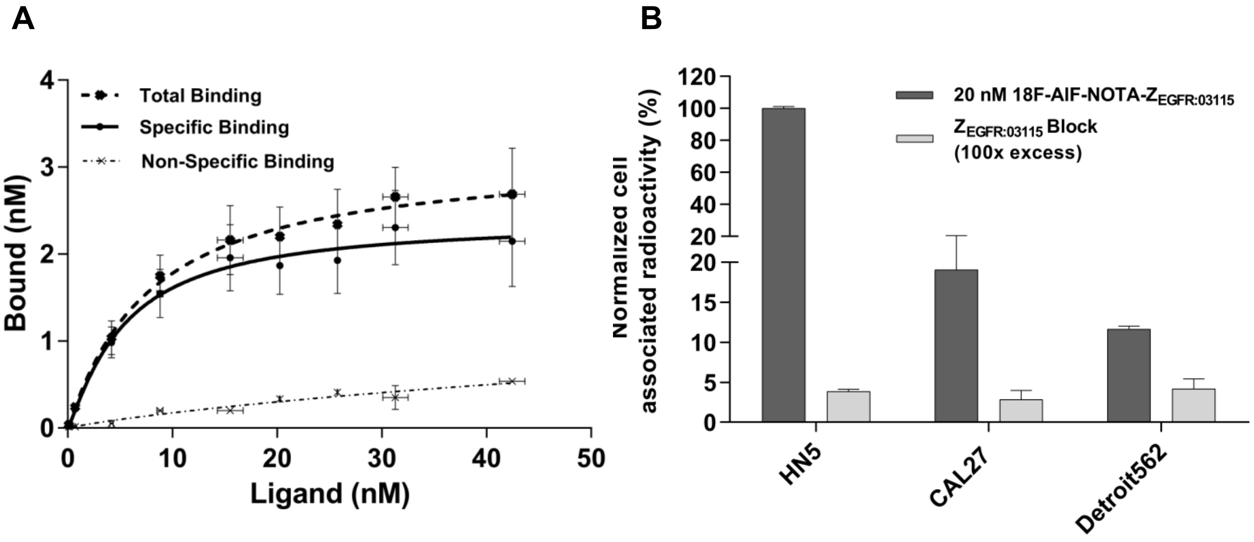

Supplemental Fig. 4: *In vitro* saturation binding assay of ^{18}F-AlF-NOTA-$Z_{EGFR:03115}$ in CAL27 cells The non-specific binding was obtained by using 100x fold excess of non-labelled Affibody molecule (**A**). *In vitro* binding specificity of ^{18}F-AlF-NOTA-$Z_{EGFR:03115}$ in HN5, CAL27 and Detroit562 cells, with and without blocking using unlabeled Affibody molecule. The results are presented as the mean of three independent experiments ± SEM.

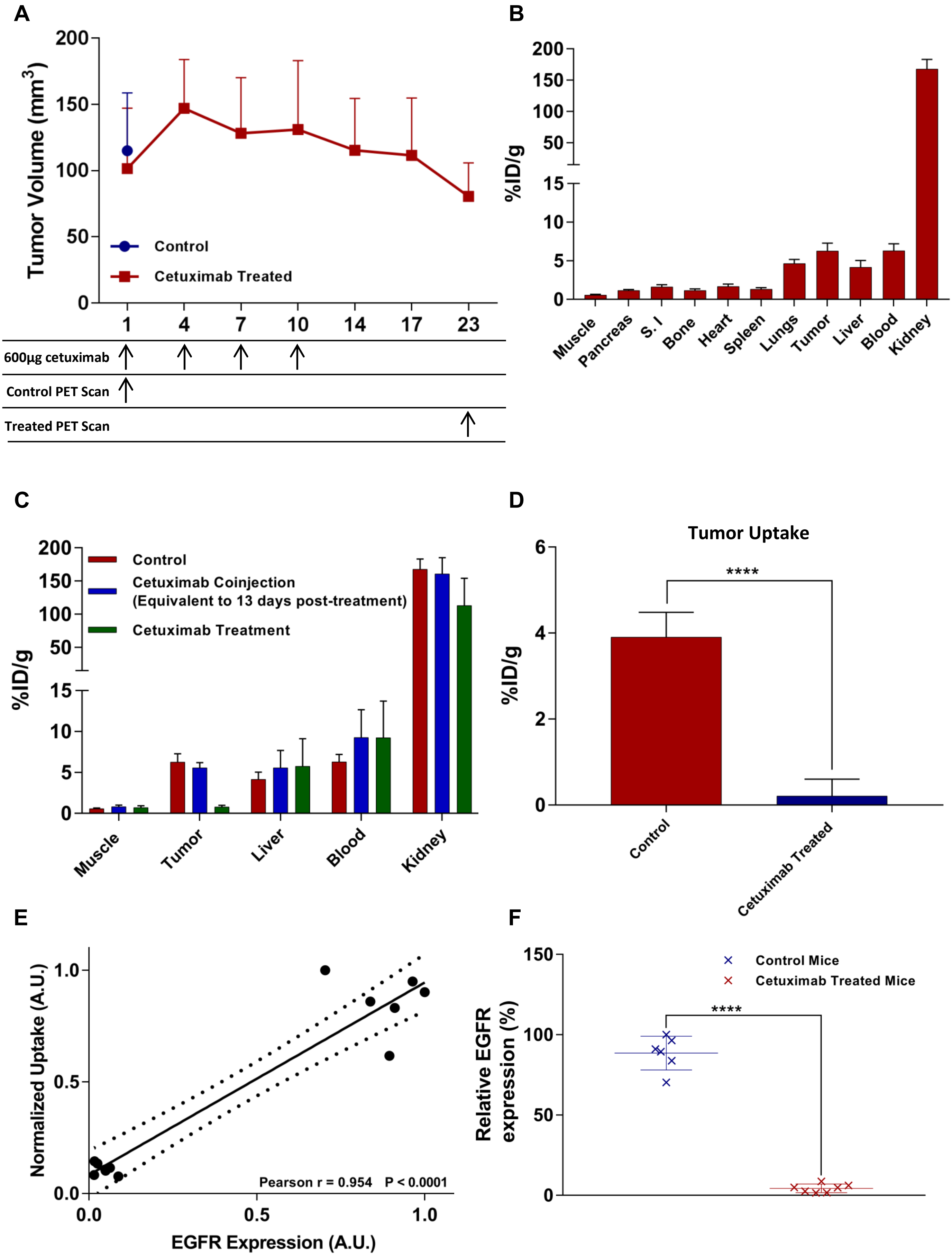
A
Tumor Volume (mm^3)
200
150
100
50
0
Control
Cetuximab Treated
1
4
7
10
14
17
23
600µg cetuximab
Control PET Scan
Treated PET Scan
B
%ID/g
200
150
100
50
15
10
5
0
Muscle
Pancreas
S. I
Bone
Heart
Spleen
Lungs
Tumor
Liver
Blood
Kidney
C
%ID/g
Control
Cetuximab Coinjection
(Equivalent to 13 days post-treatment)
Cetuximab Treatment
Muscle
Tumor
Liver
Blood
Kidney
D
Tumor Uptake
%ID/g
6
4
2
0
****
Control
Cetuximab Treated
E
Normalized Uptake (A.U.)
1.0
0.5
0.0
Pearson r = 0.954 P < 0.0001
EGFR Expression (A.U.)
F
Relative EGFR expression (%)
150
100
50
0
Control Mice
Cetuximab Treated Mice
****

G

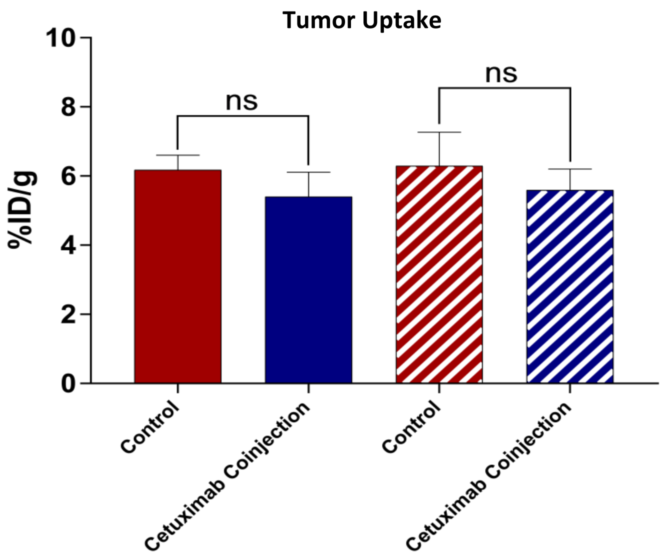

Supplemental Fig. 5:

Tumor growth curve following cetuximab treatment in mice bearing HN5 tumors. The arrows indicate the intravenous administration of cetuximab and initiation of PET scans (**A**). Biodistribution in selected organs 1 h post ^{18}F-AlF-NOTA-$Z_{EGFR:03115}$ intravenous injection in control mice (**B**). Biodistribution in selected organs 1 h post ^{18}F-AlF-NOTA-$Z_{EGFR:03115}$ intravenous injection in control mice cetuximab treated or cetuximab co-injected mice . Mice which were co-injected with a dose of cetuximab estimated to be still circulating in the system 13 days post-treatment were used to confirm the lack of interference with the radioconjugate tumor uptake (**C**). Background tissue subtracted PET quantification of treated or non-treated HN5 xenografts. (Background determined from an identical ROI on the opposite shoulder) (**D**). Spearman's rank correlation analysis for EGFR expression as determined by Western blot and the ^{18}F-AlF-NOTA-$Z_{EGFR:03115}$ tumor uptake as quantified by biodistribution. The dashed lines represent the 95% confidence levels (**E**). Relative EGFR expression determined by densitometric analysis on two separate western blots of the same HN5 tumor lysates to show the decrease in EGFR expression between control and cetuximab treated mice (**F**). ^{18}F-AlF-NOTA-$Z_{EGFR:03115}$ uptake in control HN5 tumors as assessed by PET quantification and biodistribution, in comparison to mice co-injected with a dose cetuximab estimated to be still circulating in the system 13 days post-treatment were used to confirm the lack of interference with the radioconjugate tumor uptake (**G**)

All Data in this figure are reported as mean ± SD (n = 6 - 7) (n = 3 for the cetuximab co-injected mice). Statistical significance was determined by performing a Welch's t test (*****P* < 0.0001 ns= not significant).
